# The native flora of Mountain Panachaikon (Peloponnese, Greece): new records and diversity

**DOI:** 10.1186/2241-5793-21-9

**Published:** 2014-06-03

**Authors:** Ioannis Kokkoris, Georgios Dimitrellos, Konstantinos Kougioumoutzis, Ioannis Laliotis, Theodoros Georgiadis, Argyro Tiniakou

**Affiliations:** Division of Plant Biology, Department of Biology, University of Patras, Rion, Patras, 26500 Greece

**Keywords:** Arctic-Alpine, Floristic regions, Greek endemics, NATURA 2000

## Abstract

**Background:**

This study presents the native flora of Mountain Panachaikon (N.W. Peloponnese, Greece), after extensive field work (from ~700 to 1900 m) and critical literature review.

**Results:**

The vascular native flora of Mt. Panachaikon comprises 757 taxa, 95 of which are Greek endemics, 79 are Balkan endemics, while 229 taxa are reported here for the first time. The known distribution of the Greek endemics *Alyssum montanum* subsp. *montanum* var. *graecum*, *Carum heldreichii*, *Cirsium heldreichii*, *Genista milii*, *Minuartia eurytanica* and *Seseli parnassicum* is expanded, being reported for the first time for the floristic region of Peloponnese, and the number of the known populations of the Near Threatened *Gymnospermium altaicum* subsp. *peloponnesiacum* is increased.

**Conclusions:**

The study area appears to have the second highest endemism and the highest one in W. Greece, compared with other mountains of N. Peloponnese and Sterea Ellada, while 22.10% of the endemics are protected and/or evaluated as Near Threatened to Endangered. It also exhibits a rather high proportion of Balkan endemics, in relation to its geographic location, and some genuine arctic-alpine taxa. These indicate that Mt. Panachaikon can be rendered as a plant diversity hotspot in the Peloponnese.

**Electronic supplementary material:**

The online version of this article (doi:10.1186/2241-5793-21-9) contains supplementary material, which is available to authorized users.

## Background

Southern European mountains are considered as areas of remarkably high plant diversity [1]. Especially the mountains of the Mediterranean basin exhibit high speciation rate [2] and consequently are rich in endemic species [3–5].

Mountains, without doubt, constitute the backbone of the entire Mediterranean region, as they cover ~1.7 million km^2^[6]. In fact, Greece has an intense mountainous relief, as more than 50% of its area is covered by mountains [7].

Greece is considered as one of the most biologically diverse countries of the European continent, since the Greek flora is highly diverse in relation to its size [8]. Among the 1520 taxa included in the two volumes of The Mountain Flora of Greece, 540 taxa are reported for Peloponnese [9, 10]. Peloponnese, a plant diversity hotspot [11], is rendered as a small “Cape region” [12] and the Peloponnesian mountains are considered as palaeogeographical refugia for plant taxa [13]. The importance of Peloponnesian endemic plants for the conservation of the Mediterranean and European floras is probably best expressed in that almost 1.6% of the overall Mediterranean vascular plants are endemic to Peloponnese or they are endemic to Greece and occur also in Peloponnese [12].

Peloponnese is a floristically under-explored region, since, as far as the Peloponnesian high mountains are concerned, only Mt. Killini [14, 15] and Mt. Erimanthos [16–18] can be regarded as floristically well-known. At the moment, there are no adequate data regarding the total number of taxa (endemic or not) occurring in the major mountain massifs (i.e. Mt. Chelmos, Mt. Parnon, Mt. Taygetos) of the Peloponnese. Aiming to contribute to the better knowledge of the Peloponnesian flora, in the present study we thoroughly investigated the native flora of Mt. Panachaikon. The area of the mountain studied was above the altitude of 700 m and includes the NATURA 2000 Network site “Oros Panachaiko” (GR2320007) [19, 20].

Most records of the study area’s flora are from Halàcsy [21–24], Strid [9] and Strid & Tan [10, 25, 26] and, more recently, from Phitos *et al*. [27, 28]. Several taxa were also reported by Dafis *et al*. [19, 20]. Information on some endemic taxa occurring in the study area is given by Tan & Iatrou [29]. Nevertheless, our knowledge of Mt. Panachaikon flora cannot be rendered as sufficient, as these records are fragmentary; thus, we believe that the present study can be considered as a fundamental contribution to the knowledge of Mt. Panachaikon flora, as well as to that of the Peloponnesian mountain flora. More specifically, we address the question: is Mt. Panachaikon indeed poor in endemic taxa and can it be rendered as a plant diversity hotspot in the Peloponnese?

## Results

### Flora

The vascular native flora of Mt. Panachaikon comprises 757 taxa, belonging to 370 genera and 90 families (Table 1).

**Table 1:** **Representation of vascular plant taxa in the native flora of Mt. Panachaikon**

Systematic unit	Families	Genera	Taxa	
	Nr	Nr	Nr	% of the total native flora
Pteridophytes	7	7	10	1.32
Gymnospermae	4	4	5	0.66
Dicotyledones	66	287	602	79.52
Monocotyledones	13	72	140	18.50
**Total**	**90**	**370**	**757**	**100.00**

Number of families, genera and taxa within the four major groups of vascular plants, viz. Pteridophytes, Gymnospermae, Dicotyledones and Monocotyledones, and their percentage of taxa in the native flora of Mt. Panachaikon.

The literature survey revealed 528 bibliographical records for the study area [9, 10, 19–29], in which 229 taxa were not recorded previously on Mt. Panachaikon (see Additional file 1). In total, 95 taxa are Greek endemics. New records for the study area include 14 Greek endemics and 19 Balkan endemics.

The most species-rich families of Mt. Panachaikon native flora are the *Asteraceae* (119 taxa), followed by the *Poaceae* and the *Fabaceae* (71 and 66 taxa, respectively). These three families account for more than one third of the total native flora (~34%).

Concerning the life forms (Table 2), hemicryptophytes dominate, followed by therophytes, geophytes, phanerophytes and chamaephytes.

**Table 2:** **Basic life form spectrum of the native flora of Mt. Panachaikon**

Life forms	Taxa	
	Nr	% of the total native flora
Phanerophytes	75	9.91
Chamaephytes	72	9.51
Hemicryptophytes	316	41.74
Therophytes	214	28.27
Geophytes	79	10.44
Hydrophytes	1	0.13
**Total**	**757**	**100**

The native flora of the area belongs to 15 main chorological groups (Table 3). The Mediterranean chorological group predominates; within this group, the Eurymediterranean element is dominant. The other chorological groups are represented with lower percentages.

**Table 3:** **Representation of categories of chorological groups in the native flora of Mt. Panachaikon**

Chorological group	Taxa	
	Nr	% of the total native flora
**1. Widely distributed taxa**	**241**	**31.84**
Cosmopolitan	60	7.93
Boreal	9	1.19
Tropical	8	1.06
Temperate	46	6.08
Eurasian	79	10.43
European	39	5.15
**2. Mediterranean taxa**	**342**	**45.18**
Eurymediterranean	126	16.65
Stenomediterranean	68	8.98
East-Mediterranean	55	7.27
Mediterranean- Submediterranean	86	11.36
South-Mediterranean	7	0.92
**3. Balkan taxa**	**79**	**10.43**
Balkan	53	7.00
Balkan-Italian	11	1.45
Balkan-Anatolian	15	1.98
**4. Endemic taxa**	**95**	**12.55**
**Total**	**757**	**100.00**

Bold characters indicate the main chorological groups.

### Balkan chorological group

A considerable portion of Mt. Panachaikon native flora is of Balkan origin (79 taxa; 10.43%). The vast majority of these elements do not expand beyond the Balkan Peninsula (53 taxa); 15 of the remaining taxa originate from Anatolia (all being widely distributed in Greece) and 11 taxa are also found in the circum-Adriatic countries.

### Endemism

On Mt. Panachaikon, 95 endemic taxa were found, belonging to 26 families and 66 genera, making up 12.55% of its native flora. Families rich in endemic taxa in absolute numbers are the Caryophyllaceae and the Brassicaceae (Table 4), having a proportion of endemism (27.45%, and 30.55%, respectively) higher than that of the total native flora (12.55%).

**Table 4:** **Representation of Greek endemic taxa within the families of the native flora of Mt. Panachaikon**

Family	Greek endemic taxa	
	Nr	% within the family
Caryophyllaceae	14	27.45
Asteraceae	12	10.08
Brassicaceae	11	30.55
Apiaceae	6	17.65
Fabaceae	6	9.23
Rubiaceae	6	28.57
Lamiaceae	5	15.15
Scrophulariaceae	5	22.73
Boraginaceae	4	20.00
Poaceae	4	5.63
Iridaceae	3	42.86
Amaryllidaceae	2	25.00
Asparagaceae	2	14.28
Campanulaceae	2	25.00
Liliaceae	2	33.33
Araceae	1	100.00
Berberidaceae	1	100.00
Colchicaceae	1	100.00
Crassulaceae	1	16.67
Dipsacaceae	1	16.67
Geraniaceae	1	11.11
Orchidaceae	1	14.29
Pinaceae	1	100.00
Plantaginaceae	1	16.67
Ranunculaceae	1	4.76
Violaceae	1	14.29

The majority (64.21%) of the endemic taxa found on Mt. Panachaikon distribute to three or more floristic regions, followed by those found in the floristic regions of Sterea Ellada and/or Peloponnese (see Additional file 2). Among the 95 Greek endemic taxa, *Alyssum montanum* subsp. *montanum* var. *graecum*, *Carum heldreichii*, *Genista milii*, *Cirsium heldreichii*, *Minuartia eurytanica* and *Seseli parnassicum* are the most interesting members of this chorological group as they are found for the first time, not only on Mt. Panachaikon, but also in the floristic region of Peloponnese. In fact, until recently *Carum heldreichii* was thought to occur only in the floristic region of Sterea Ellada and *Seseli parnassicum* only in the floristic regions of Sterea Ellada and South Pindos. Additionally, *Alyssum montanum* subsp. *montanum* var. *graecum* and *Genista milli* were thought to occur only in the floristic regions of Sterea Ellada and the West Aegean islands, with *Genista milli* presenting its southernmost populations at Mt. Vardousia [30].

A very interesting record is that of the Peloponnesian endemic taxon *Gymnospermium altaicum* subsp. *peloponnesiacum*, which is evaluated as Near Threatened [31]; Mt. Panachaikon harbours the northernmost populations of this taxon within its total distribution range and we have discovered additional sub-populations, to the already known [29, 31, 32] occurring in the mountain, which are much larger in size. More specifically, we have recorded ~4000 individuals of *Gymnospermium altaicum* subsp. *peloponnesiacum* from five new localities in the study area.

In total, 45 out of 95 endemic taxa are under some protection and/or extinction risk status (see Additional file 2). More specifically, *Ophrys argolica* is included in the CITES Convention [33] and in the European Council Directive 92/43/EEC [34], three taxa are included in the Bern Convention [35], 19 taxa in the Greek Presidential Decree 67/1981 [36], 43 taxa in the IUCN Red Lists [37–39], three taxa in the European Red List of Vascular Plants [40] and finally four taxa in the Red Data Books of Rare and Threatened Plants of Greece [27, 28]. It must be noticed, that the protected taxa as well as those evaluated as Near Threatened or Vulnerable correspond to 22.10% (21 taxa) of the study area’s endemic flora.

### Arctic-Alpine taxa and other records

The three widespread arctic-alpine taxa, already known from the study area, were found growing on siliceous soils at 1500–1700 m, on the above the tree line grasslands (*Luzula spicata* and *Phleum alpinum*) and rock crevices (*Saxifraga adscendens* subsp. *parnassica*).

It must also be mentioned, that the non endemic taxa *Silene roemeri* and *Vicia canescens* are reported for the first time from the floristic region of Peloponnese, thus expanding their distribution areas.

## Discussion

The floristic character of Mt. Panachaikon is probably altered, as a result of the strong human impacts present in the study area; the intense grazing regime, the road construction works, as well as the close proximity to a large city (Patras) could explain the high amount of Cosmopolitan elements (7.93%). However, the predominance of the Mediterranean chorological group (45.18%) highlights the geographical position and the climatic characteristics of the study area; the predominance of hemicryptophytes (41.74%) indicates the intense mountainous character of the flora, while the high percentage of therophytes (28.27%) reflects its Mediterranean character.

The most species-rich genera in Peloponnese are *Trifolium*, *Silene*, *Allium*, *Ranunculus*, *Galium*, *Euphorbia* and *Astragalus*[29]. This pattern also seems to be true in the study area, although with some modifications (*Crepis* and *Veronica* are more abundant than *Galium*).

Previous studies suggested that the representation of the Balkan endemic chorological group gradually decreases in Sterea Ellada and the Peloponnese, whilst in Crete it is represented at a minimal scale [41]. Our results are in accordance with Strid [41], as on Mt. Panachaikon the Balkan chorological group is represented with higher percentage than on Mt. Erimanthos [17, 18] or on Mt. Killini [15], two mountains located at lower latitudes than the study area and with lower percentage than on other Greek mountains located at higher latitudes, i.e. Mt. Iti [42] and Mt. Timfristos [43]. Regarding the Balkan-Italian element, our findings support Strid [44], indicating a decreased representation in southern parts of Greece, since connections between the Greek and Italian mountain floras clearly follow a northern, Adriatic route. As for the Balkan-Anatolian element, our results are in accordance with Strid [45], indicating a strong decrase in its representation towards Peloponnese.

A similar trend is observed in the arctic-alpine elements in the Scardo-Pindhic mountain system, where according to Stevanović *et al.*[46], their number sharply decreases in a southward direction. Strictly arctic-alpine taxa are rare in Greece, and generally restricted to some of the highest mountain tops in the northern parts of the country [44]. Relatively few arctic-alpine taxa occur above 1500 m on all the high mountains of N. Peloponnese and this phenomenon is attributed to the unfavorable survival conditions of the arctic-alpine flora on those mountains, to the absence of strong glaciation during the Pleistocene, as well as to their distance from the main centers of Balkan glacial flora [46]. Nevertheless, the representation of this chorological group on Mt. Panachaikon is rendered significant, given the study area’s relatively low altitude, geographical location and geological composition, since according to Stevanović *et al.*[46], siliceous or predominantly siliceous mountains have more arctic-alpine taxa than limestone or predominantly limestone ones. The three arctic-alpine taxa occuring in the study area were found on siliceous soils derived from the selective solution and outwashing (due to high precipitation) of the carbonate part of the limestone substrate, as a result of the extremely rapid weathering and erosional processes [47] occurring in the high altitudes where these taxa are found. The number of the Balkan endemic and arctic-alpine taxa decreases in a north to south axis in Greece, while a reverse trend is observed for the Greek endemics [41]. Thus, the proportion of Balkan endemics to Greek endemics found on Mt. Panachaikon is reasonable to be quite high (1:1.2), since the study area is located almost in the middle of this axis.

According to Tan & Iatrou [29], 2960 taxa are found in the Peloponnese, 355 of which are considered endemics (12%). Peloponnesian mountains maintain higher endemic species richness compared to lowland areas [12]; Mt. Panachaikon hosts 95 endemic taxa (12.55%), nearly three times the taxa Methana peninsula hosts (35 taxa) [48]. The high proportion of endemic taxa of Caryophyllaceae (27.45%) and Brassicaceae (30.55%) agrees with the trend observed in the whole Greek endemic flora [8]. The total number of endemic taxa present in the study area is high compared to the total, especially when one takes into consideration the relatively low altitude of Mt. Panachaikon (1926 m), its geographic position (close proximity to a large city and on the edge of two floristic regions of Greece, namely Peloponnese and Sterea Ellada) and the intensity of the human-induced disturbance. Furthermore, compared to the levels of endemism in Mt. Erimanthos (9.39%) [17, 18], Mt. Killini (13.20%) [15], Mt. Timfristos (7.48%) [43], Mt. Vardousia (9.40%) [30, 49], Mt. Iti (6.70%) [42] and Mt. Elikon (9.16%) [50], mountains neighbouring Panachaikon with larger size and higher altitudes (except Mt. Elikon, 1748 m), the level of endemism in the study area is remarkably high and actually appears to be the second highest among the examined mountains (Table 5); apparently, it is the highest in western Greece. Thus, Mt. Panachaikon constistutes an endemic hotspot in Peloponnese and this finding complements the analysis of Trigas *et al*. [12], according to which the N. Peloponnese mountains (Mt. Killini, Mt. Chelmos and secondarily Mt. Erimanthos) represent an endemic hotspot in this region. The new data presented herein, could lead to the inclusion of Mt. Panachaikon in the priority conservation areas in Peloponnese, as proposed by Trigas *et al.*[12].

**Table 5:** **Levels of endemism**

Region	Greek endemic taxa	
	Nr	%
Peloponnese	355	12.00
Panachaikon	95	12.55
Erimanthos	90	9.39
Killini	134	13.20
Timfristos	90	7.48
Vardousia	105	9.40
Iti	77	6.70
Elikon	116	9.16

Endemism in the floristic region of Peloponnese and in the mountains Panachaikon, Erimanthos, Killini, Timfristos, Vardousia, Iti and Elikon.

The existence of biregional endemics is a good indication of phytogeographical connections between regions [8]; consequently, nearly one fifth (18 taxa) of the endemic taxa present in the study area provide valuable information regarding its phytogeographical position. As it was expected, Mt. Panachaikon shows higher affinities with the floristic region of Sterea Ellada, since, according to Georghiou & Delipetrou [8], the floristic region of Peloponnese is chorologically closer connected to the floristic region of Sterea Ellada than any other floristic region and according to Strid [44], the Gulf of Corinth scarcely appears as a phytogeographical barrier at all. Our results concur with the above mentioned findings, since 15 biregional endemics present on Mt. Panachaikon occur exclusively in the floristic regions of the Peloponnese and Sterea Ellada.

## Conclusions

Mt. Panachaikon despite its relatively small size and low altitude, is characterised by a unique flora, even though it lies near a weak biogeographical barrier and has suffered from intense human impacts; it demonstrates a high level of endemism and has a rather strong “Peloponnesian” character, as 16 taxa are found exclusively in the floristic region of Peloponnese and it appears to be a meeting point of several migration routes of different directions. This phenomenon is apparent, since Mt. Panachaikon harbours the northernmost populations of *Gymnospermium altaicum* subsp. *peloponnesiacum* and the southernmost populations of *Allysum montanum* subsp. *montanum* var. *graecum*, *Carum heldreichii*, *Genista milii, Cirsium heldreichii*, *Minuartia eurytanica, Seseli parnassicum*, *Silene roemeri* and *Vicia canescens*.

Finally, among the endemic taxa present on Mt. Panachaikon, 10 are considered to be at extinction risk (the Vulnerable *Erodium chrysanthum*, *Ophrys argolica* and *Peucedanum achaicum*, the Rare *Arabis subflava*, *Arenaria guicciardii*, *Dianthus androsaceus*, *Genista milli*, *Scutellaria rupestris* and *Seseli parnasicum* and the Near Threatened *Gymnospermium altaicum* subsp. *peloponnesiacum*). New records of sub-populations for the Near Threatened taxon *Gymnospermium altaicum* subsp. *peloponnesiacum*, enhance the need of an extensive research for all taxa considered to be under an extinction risk status. For all these taxa, their population trend is unknown and no conservation measures have been taken for their protection. Therefore, it is necessary to estimate and monitor their population size and dynamics, in order to effectively protect them.

## Methods

### Study area

**Figure 1:**
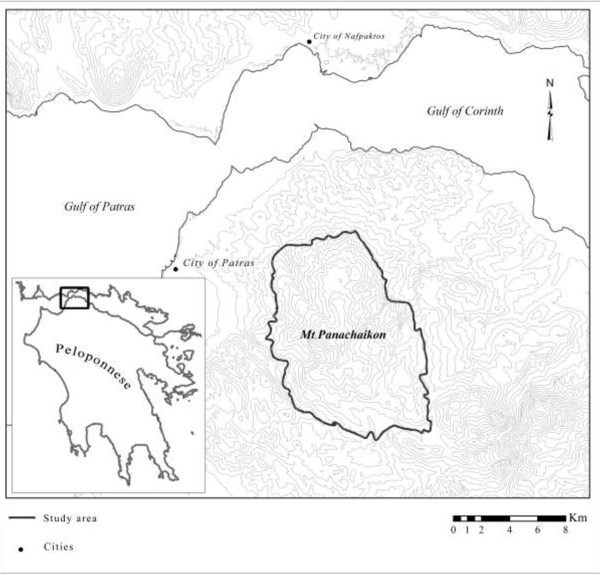
**Map of the study area.** Topographic map of North Peloponnese-Greece, indicating the study area of Mt. Panachaikon (1926 m).

Mt. Panachaikon is located at the northwestern part of the floristic region of Peloponnese, east-southeast of the city of Patras (Figure 1). It is characterized by a multifarious relief with many different inclinations and exposures and has numerous peaks, the highest being Voidias (1926 m). Geotectonically, Mt. Panachaikon constitutes a representative part of the Olonos-Pindhos geotectonic zone [51–53]. It is characterized by a variety of geological substrates and soil types, comprising sediments of the alpine orogenetic belt (limestones, schists, flysh rocks and radiolarites) that are strongly fractured, as well as Pliocene and Quaternary formations of alternating layered sediments (marls, clays, conglometates, gravel). Numerous gorges and streams interrupt the latter formations and control the study area’s hydrological conditions [54].

Mt. Panachaikon belongs to the humid bioclimatic zone with a severe to cold winter and has a bioclimatic character of weak mid-Mediterranean to sub-Mediterranean type at higher altitudes [55–58]. The biologically arid days at lower altitudes during the summer season are 40–75 and at higher altitudes 0–40. The coldest month’s mean temperature is 3°C. At the western and southwestern parts of Mt. Panachaikon, the annual precipitation exceeds 1500 mm, while at the rest mountain parts it ranges from 1350 mm to 1500 mm [54].

### Floristic inventory & evaluation

Several collection and field observation trips to the study area were performed between spring 2008 and autumn 2012, within the framework of the first author’s PhD thesis, in order to acquire an integrated knowledge of Mt. Panachaikon flora and vegetation. In total, we have spent 92 days in the field and visited 452 different collection sites at altitudes ranging from ~700 m to 1900 m, covering all habitat and soil types present on the study area; the sites reported by Laliotis in 2001 (unpublished data) were revisited and re-sampled. Herbarium specimens are deposited at the Botanical Museum of the University of Patras (UPA). Plant identification is according to Tutin *et al.*[59], Strid [9] and Strid & Tan [10, 25, 26]. Plant nomenclature is according to Greuter *et al*. [60] and Greuter & Raab-Straube [61]. For family delimitation, we followed APG III [62]. The nomenclature, status and geographical distribution in the floristic regions of Greece of the endemic taxa are based on Tan & Iatrou [29] and Georgiou & Delipetrou [8]. We have excluded from the flora list and floristic analysis the alien taxa occurring in the study area; their alien status was determined according to Arianoutsou *et al*. [63]. The status of the arctic-alpine taxa present on Mt. Panachaikon is according to Stevanović *et al.*[46]. The protection status of Mt. Panachaikon endemic taxa is according to CITES [33], Bern Convention [35], Hellenic Official Government Gazette [36] and The Council of the European Communities [34]. The extinction risk status of Mt. Panachaikon endemic taxa is according to IUCN [37, 38], Walter & Gillett [39], Phitos *et al*. [25, 26] and Bilz *et al*. [40]. The life form categories follow Raunkiaer [64], while Pignatti’s [65] classification was used for the chorological analysis.

## Authors’ information

IK, forester, MSc, is a PhD candidate interested in plant systematics, biogeography and ecology, targeting his research on conservation, remote sensing, monitoring and management of terrestrial ecosystems.

GD, forester, PhD, is a research associate at the University of Patras, working on subjects of plant systematics, ecology, conservation and biogeography.

KK, biologist, MSc, is a PhD candidate with a keen interest in the biogeography and plant diversity of the South Aegean Volcanic Arc and the Aegean Islands, as well as in the local drivers of diversity regarding different types of habitat islands.

IL, biologist, MSc, is an active field botanist and a biology high school teacher, with interest in botany, plant taxonomy, ecology and biogeography.

TG is a Professor Emeritus at the University of Patras, working for many years on subjects relative with the Greek flora and vegetation, plant systematics, ecology, management and conservation of terrestrial ecosystems.

AT is an Assistant Professor at the University of Patras, working for many years on subjects relative with the Greek flora and has special interest on plant taxonomy, as well as on the monitoring, management and conservation of land and wetland ecosystems.

## Electronic supplementary material

Additional file 1: **New records from the native flora of Mt. Panachaikon.** (DOC 93 KB)

Additional file 2: **Protection status and extinction risk designation of the Greek endemic taxa of Mt. Panachaikon and their geographical distribution in the floristic regions of Greece.** (DOC 232 KB)
