# Supplementary material for: The native flora of Mountain Panachaikon (Peloponnese, Greece): new records and diversity
Source: J Biol Res (Thessalon). 2014 Jun 3;21(1):9. doi: 10.1186/2241-5793-21-9 (PMC4389996; doi:10.1186/2241-5793-21-9)
Supplement: Supplementary file 1 — Additional file 1: New records from the native flora of Mt. Panachaikon. (DOC 93 KB) [file 40709_2013_7_MOESM1_ESM.doc]

**Additional file 1.** New records from the native flora of Mt. Panachaikon.

Only taxa new to the investigated area appear in the catalogue given below.

Abbreviations used:

JK: Ioannis Kokkoris vouchers

Obs.: Field observations

JL: Ioannis Laliotis (unbublished records)

New records’ collection dates:

(a): 29/06/2008-06/07/2008

(b): 13/06/2009-20/07/2009

(c): 04/06/2010

(d): 28/06/2011

(e): 18/07/2011-26/07/2011

(f): 10/07/2012

New records’ collection sites:

**1**. 870m. N 38° 15´ 49.7˝ E 21° 53´ 13.9˝

**2**. 970m. N 38° 16´ 0.8˝ E 21° 52´ 42.5˝

**3**. 1240m. N 38° 15´ 41.4˝ E 21° 52´ 17.3˝

**4**. 1330m. N 38° 15´ 29.5˝ E 21° 52´ 20.4˝

**5**. 1435m. N 38° 15´ 5.5˝ E 21° 52´ 0.5˝

**6**. 1510m. N 38° 14´ 46.4˝ E 21° 51´ 55.1˝

**7**. 1490m. N 38° 14´ 47.3˝ E 21° 51´ 58.1˝

**8**. 1510m. N 38° 14´ 39.5˝ E 21° 52´ 7.7˝

**10**.1580m. N 38° 14´ 23.1˝ E 21° 51´ 58.0˝

**11**. 1550m. N 38° 14´ 23.1˝ E 21° 51´ 46.3˝

**12**. 1510m. N 38° 14´ 32.2˝ E 21° 52´ 19.6˝

**13**. 1520m. N 38° 14´ 33.4˝ E 21° 52´ 13.1˝

**14**. 1410m. N 38° 15´ 1.9˝ E 21° 51´ 56.3˝

**15**. 990m. N 38° 14´ 10.5˝ E 21° 49´ 40.8˝

**16**. 1080m. N 38° 13´ 46.2˝ E 21° 49´ 52.4˝

**17**. 1230m. N 38° 13´ 29.9˝ E 21° 50´ 16.1˝

**18**. 1405m. N 38° 12´ 42.5˝ E 21° 50´ 45.1˝

**19**. 1460m. N 38° 12´ 48.9˝ E 21° 50´ 55.5˝

**20**. 1495m. N 38° 12´ 47.0˝ E 21° 51´ 2.2˝

**21**. 1510m. N 38° 12´ 47.8˝ E 21° 51´ 6.3˝

**22**. 1625m. N 38° 12´ 44.3˝ E 21° 51´ 18.4˝

**23**. 1660m. N 38° 12´ 42.8˝ E 21° 51´ 19.3˝

**24**. 1665m. N 38° 12´ 43.5˝ E 21° 51´ 21.5˝

**25**. 1670m. N 38° 12´ 46.2˝ E 21° 51´ 20.8˝

**26**. 1645m. N 38° 12´ 45.6˝ E 21° 51´ 31.1˝

**27**. 1670m. N 38° 12´ 35.8˝ E 21° 51´ 32.7˝

**28**. 1735m. N 38° 12´ 14.9˝ E 21° 51´ 53.5˝

**29**. 1730m. N 38° 12´ 15.2˝ E 21° 51´ 53.6˝

**30**. 1725m. N 38° 12´ 13.8˝ E 21° 51´ 57.1˝

**32**. 1725m. N 38° 12´ 13.7˝ E 21° 51´ 57.7˝

**34**. 1730m. N 38° 12´ 12.6˝ E 21° 51´ 56.1˝

**35**. 1730m. N 38° 12´ 8.9´´ E 21° 51´ 55.3´´

**37**. 1720m. N 38° 12´ 29.3˝ E 21° 52´ 23.8˝

**38**. 800m. N 38° 14´ 59.2˝ E 21° 54´ 16.5˝

**40**. 680m. N 38° 16´ 24.3˝ E 21° 50´ 51.8˝

**41**. 830m. N 38° 15´ 59.7˝ E 21° 50´ 40.1˝

**43**. 1065m. N 38° 15´ 41.6˝ E 21° 51´ 8.3˝

**44**. 840m. N 38° 9´ 14.9˝ E 21° 51´ 3.7˝

**45**. 900m. N 38° 9´ 5.3˝ E 21° 51´ 34.8˝

**46**. 1015m. N 38° 8´ 36.9˝ E 21° 53´ 11.9˝

**47**. 900m. N 38° 8´ 50.7˝ E 21° 53´ 49.4˝

**48**. 1010m. N 38° 8´ 44.2˝ E 21° 54´ 44.9˝

**49**. 1020m. N 38° 8´ 44.8˝ E 21° 54´ 49.9˝

**51**. 1020m. N 38° 8´ 44.2˝ E 21° 54´ 47.2˝

**52**. 1160m. N 38° 9´ 9.2˝ E 21° 56´ 50.8˝

**53**. 1000m. N 38° 8´ 40.3˝ E 21° 54´ 31.8˝

**54**. 1020m. N 38° 12´ 8.9´´ E 21° 51´ 55.3´´

**55**. 1300m. N 38° 13´ 4.9˝ E 21° 50´ 29.9˝

**58**. 1700m. N 38° 12´ 47.6˝ E 21° 51´ 5.3˝

**61**. 1615m. N 38° 12´ 39.6˝ E 21° 51´ 11.4˝

**62**. 1650m. N 38° 11´ 33.2˝ E 21° 52´ 5.1˝

**63**. 1680m. N 38° 12´ 38.5˝ E 21° 51´ 15.4˝

**64**. 1480m. N 38° 12´ 49.3˝ E 21° 50´ 53.9˝

**66**. 1450m. N 38° 9´ 53.1˝ E 21° 53´ 17.1˝

**67**. 1250m. N 38° 13´ 41.6˝ E 21° 50´ 14.3˝

**68**. 1400m. N 38° 12´ 38.2˝ E 21° 50´ 13.8˝

**69**. 1620m. N 38° 13´ 26.9˝ E 21° 50´ 57.3˝

**70**. 1700m. N 38° 12´ 45.4˝ E 21° 51´ 21.5˝

**71**. 1700m. N 38° 11´ 33.0˝ E 21° 51´ 55.9˝

**73**. 990m. N 38° 12´ 10.0˝ E 21° 51´ 55.8˝

**74**. 1500m. N 38° 16´ 4.1˝ E 21° 52´ 33.8˝

**75**. 790m. N 38° 14´ 17.2˝ E 21° 51´ 54.4˝

**76**. 1490m. N 38° 10´ 36.9˝ E 21° 49´ 41.2˝

**81**. 1120m. N 38° 14´ 9.9˝ E 21° 54´ 2.4˝

**82**. 1120m. N 38° 14´ 3.6˝ E 21° 54´ 0.0˝

**83**. 1120m. N 38° 9´ 36.6˝ E 21° 53´ 22.1˝

**84**. 1120m. N 38° 9´ 35.6˝ E 21° 52´ 50.8˝

**85**. 1150m. N 38° 9´ 29.2˝ E 21° 54´ 15.4˝

**87**. 1020m. N 38° 8´ 45.3˝ E 21° 52´ 38.6˝

**89**. 1700m. N 38° 9´ 3.5˝ E 21° 52´ 32.3˝

**90**. 1700m. N 38° 12´ 13.6˝ E 21° 51´ 4.8˝

**91**. 1700m. N 38° 12´ 14.1˝ E 21° 51´ 3.8˝

**92**. 1700m. N 38° 12´ 15.5˝ E 21° 51´ 3.3˝

**93**. 1780m. N 38° 11´ 37.8˝ E 21° 52´ 16.1˝

**95.** 1600m. N 38° 12´ 28.5˝ E 21° 50´ 58.9˝

**96**. 800m. N 38° 15´ 41.0˝ E 21° 50´ 10.2˝

**97**. 950m. N 38° 11´ 20.4˝ E 21° 49´ 36.5˝

**101**. 680m. N 38° 12´ 36.8˝ E 21° 48´ 52.8˝

**106**. 1300m. N 38° 12´ 29.6˝ E 21° 50´ 14.0˝

**108**. 680m. N 38° 15´ 31.6˝ E 21° 49´ 14.5˝

**109**. 1000m. N 38° 12´ 53.1˝ E 21° 49´ 56.9˝

**110.** 1450m. N 38° 13´ 57.4˝ E 21° 51´ 9.3˝

**111**. 900m. N 38° 11´ 28.1˝ E 21° 50´ 8.7˝

**112**. 1400m. N 38° 12´ 6.1˝ E 21° 50´ 35.7˝

**114**. 1100m. N 38° 9´ 59.1˝ E 21° 54´ 22.6˝

**115**. 700m. N 38° 11´ 14.3˝ E 21° 49´ 59.5˝

**118**. 600m. N 38° 15´ 40.0˝ E 21° 49´ 23.8˝

**119**. 700m. N 38° 12´ 40.3˝ E 21° 49´ 15.9˝

**120**. 1300m. N 38° 9´ 30.6˝ E 21° 54´ 29.2˝

**122**. 1500m. N 38° 12´ 41.7˝ E 21° 50´ 55.5˝

**124**. 1300m. N 38° 13´ 3.6˝ E 21° 50´ 37.3˝

**126**. 1300m. N 38° 9´ 24.4˝ E 21° 54´ 56.8˝

**127**. 1310m. N 38° 9´ 46.7˝ E 21° 53´ 31.9˝

**128**. 700m. N 38° 12´ 36.5˝ E 21° 48´ 56.9˝

**129**. 800m. N 38° 13´ 14.2˝ E 21° 49´ 12.9˝

**131**. 1140m. N 38° 9´ 54.2˝ E 21° 54´ 19.4˝

**132**. 1000m. N 38° 8´ 23.5˝ E 21° 54´ 14.6˝

**133**. 1450m. N 38° 12´ 24.5˝ E 21° 50´ 31.4˝

**135**. 700m. N 38° 12´ 47.8˝ E 21° 49´ 21.7˝

**136**. 800m. N 38° 8´ 46.4˝ E 21° 54´ 12.6˝

**137**. 700m. N 38° 12´ 13.8˝ E 21° 48´ 18.9˝

**138**. 1200m. N 38° 15´ 45.4˝ E 21° 52´ 43.7˝

**139**. 1300m. N 38° 13´ 20.4˝ E 21° 50´ 42.7˝

**140**. 1400m. N 38° 13´ 19.1˝ E 21° 51´ 7.7˝

**141**. 1040m. N 38° 9´ 41.1˝ E 21° 54´ 16.9˝

**155**. 1000m. N 38° 13´ 52.6˝ E 21° 50´ 9.9˝

**157**. 500m. N 38° 15´ 33.3˝ E 21° 49´ 13.1˝

**158**. 680m. N 38° 15´ 33.5˝ E 21° 49´ 22.4˝

**160**. 1100m. N 38° 8´ 57.2˝ E 21° 54´ 44.2˝

**161**. 700m. N 38° 12´ 39.4˝ E 21° 48´ 58.9˝

**164**. 870m. N 38° 15´ 40.5˝ E 21° 53´ 13.6˝

**165**. 1100m. N 38° 13´ 49.8˝ E 21° 50´ 5.3˝

**166**. 1000m. N 38° 10´ 4.3˝ E 21° 51´ 9.7˝

**167**. 1000m. N 38° 8´ 33.6˝ E 21° 54´ 5.4˝

**168**. 1100m. N 38° 9´ 2.1˝ E 21° 54´ 41.4˝

**169**. 1000m. N 38° 13´ 55.9˝ E 21° 49´ 28.5˝

**170**. 1200m. N 38° 15´ 44.2˝ E 21° 52´ 25.1˝

**171**. 1300m. N 38° 10´ 26.4´´ E 21° 54´ 33.3´´

**173**. 1140m. N 38° 10´ 19.0˝ E 21° 56´ 23.6˝

**174**. 780m. N 38° 16´ 24.9˝ E 21° 52´ 27.6˝

**180**. 1200m. N 38° 15´ 41.9˝ E 21° 52´ 26.5˝

**181**. 1200m. N 38° 13´ 38.6˝ E 21° 50´ 43.5˝

**182**. 700m. N 38° 15´ 11.9˝ E 21° 49´ 49.6˝

**183**. 1100m. N 38° 13´ 45.2˝ E 21° 49´ 48.6˝

**188**. 1200m. N 38° 9´ 13.2˝ E 21° 54´ 40.3˝

**189**. 1700m. N 38° 12´ 47.1˝ E 21° 52´ 13.6˝

**190**. 1200m. N 38° 15´ 16.8˝ E 21° 52´ 0.9˝

**191**. 1600m. N 38° 14´ 13.6˝ E 21° 51´ 41.1˝

**192**. 910m. N 38° 11´ 21.7´´ E 21° 50´ 23.7´´

**196**. 1440m. N 38° 13´ 22.2˝ E 21° 50´ 57.7˝

**197**. 885m. N 38° 15´ 45.5˝ E 21° 50´ 34.1˝

**198**. 900m. N 38° 8´ 48.2˝ E 21° 54´ 25.2˝

**199**. 1100m. N 38° 14´ 3.2˝ E 21° 49´ 54.4˝

**200**. 700m. N 38° 16´ 20.4˝ E 21° 52´ 56.0˝

**201**. 1500m. N 38° 12´ 34.7˝ E 21° 50´ 50.1

**202**. 1140m. N 38° 14´ 5.0˝ E 21° 50´ 29.8˝

**300**. 1700m. N 38° 10´ 43.7˝ E 21° 51´ 18.4˝

**301**. 1600m. N 38° 10´ 43.1˝ E 21° 52´ 23.9˝

**302**. 1250m. N 38° 13´ 38.3˝ E 21° 50´ 41.6˝

**303**. 1752m. N 38° 12´ 9.3´´ E 21° 51´ 52.5´´

**304**. 1780m. N 38° 12´ 10.9˝ E 21° 51´ 57.3˝

**305**. 1680m. N 38° 10´ 51.9˝ E 21° 51´ 56.4˝

**307**. 1380m. N 38° 12´ 48.5˝ E 21° 50´ 36.9˝

**308**. 1810m. N 38° 12´ 10.2˝ E 21° 51´ 17.4˝

**309**. 1810m. N 38° 12´ 8.6˝ E 21° 51´ 42.8˝

**311**. 1700m. N 38° 12´ 28.7˝ E 21° 52´ 24.8˝

**312**. 1910m. N 38° 12´ 0.5˝ E 21° 52´ 8.0˝

**313**. 1700m. N 38° 12´ 9.2 ˝ E 21° 52´ 58.6 ˝

**315**. 750m. N 38° 16´ 22.1˝ E 21° 50´ 52.5˝

**316**. 1450m. N 38° 14´ 46.0˝ E 21° 51´ 47.1˝

**318**. 840m. N 38° 15´ 32.7˝ E 21° 50´ 33.2˝

**343**. 1590m. N 38° 12´ 2.4˝ E 21° 51´ 1.4˝

**344**. 755m. N 38° 15´ 45.2˝ E 21° 50´ 6.2˝

**352**. 1530m. N 38° 14´ 37.7˝ E 21° 52´ 8.9˝

**354**. 1310m. N 38° 11´ 34.8˝ E 21° 50´ 45.9˝

| Life forms: |  |
| --- | --- |
| *Therophytes* (T)  Tcaesp T. caespitose  Tpar T. parasite  Trept T. reptant  Tscap T. scapose  *Geophytes* (G)  Gbulb G. bulbous  Grhiz G. rhizomatous  *Hemicryptophytes* (Η)  H bienn H. biennial  Hcaesp Η. caespitose  Hrept H. reptant  Hros Η. rosulate  Hscap Η. scapose | *Chamaephytes* (Ch)  Chfrut Ch. fruticose  Chpulv Ch. pulvinate  Chrept Ch. reptant  Chsucc Ch. succulent  Chsuffr Ch. suffruticose  *Phanerophytes* (P)  Pcaesp P. caespitose  Pep P. epiphytic  Plian P. lianose  Pscap P. scapose  *Mega-phanerophytes* (MP)  *Nano-phanerophytes* (NP) |

| Chorological groups: |  |
| --- | --- |
| **Widely distributed taxa**:  Cosmopolitan (Subcosmop., Cosmop.)  Pantropics (Pantrop.)  Paleotemperate (Paleotemp.)  Boreal (Circumbor.)  Eurasian (Euras., E-Med.-Asiat, Europ.-S-Sib., Eurosib., S-Europ.-Subsib., Orof.-W-Eurasiat.)  European-Caucasian (C-Europ.-Caucas., Europ.-Caucas.)  European (Centro-Europ., E-Europ., SE-Europ., S-Europ., Orof.-S-Europ.)  **Mediterranean taxa**  Mediterranean (Med.)  South-Mediterranean (S-Med.)  Ε. Mediterranean (C-E-Med., C-Med.-Orient., Ε-Med., E-Med.-Mont., NE-Med., NE-Med.-Mont.)  Western-Mediterranean (W-Med.) Eurymediterranean (Εurymed., Eurymed.-Orient.) | Stenomediterranean (Stenomed.)  Eastern-Stenomediterranean (E-Stenomed., Stenomed.-Orient. Stenomed.-Nordorient.)  Mediterranean-Atlantic (Med.-Atl.)  Mediterranean - Submediterranean (C-Europ.-Submed., Euras.-Subozean.-Med., Med.-Submed., Eurymed.-Subsib., Pont., S-Europ.-Pont., Submed.-Euras.)  Mediterranean-Caucasian (E-Med.-Caucas.)  Mediterranean - Turanian [(Eury)-Med.-Turan., Med.-Turan., NE-Med.-Turan.]  Eurymediterranean - Turanian (Eurymed.-Turan.)  **Balkan** (Balkan, Balkan-An., Balkan-It.)  **Endemic** (Endemic) |

Ferns and Fern Allies

*Aspleniaceae*

*Asplenium ceterach* L. - Hros, Paleotemp.; 2, (a), JK21; 44, (a), JK131.

*Asplenium trichomanes* L. - Hros, Cosmop.; 112, (a), JK278.

*Cystopteridaceae*

*Cystopteris fragilis* (L.) Bernh. - Hcaesp, Cosmop.; 24, (a), JK75.

*Dennstaedtiaceae*

*Pteridium aquilinum* (L.) Kuhn - Grhiz, Cosmop.; 48, (a), JK193; 54, (a), JK140.

*Equisetaceae*

*Equisetum palustre* L. - Grhiz, Circumbor.; 40, (a), JK104.

*Pteridaceae*

*Adiantum capillus*-*veneris* L. - Grhiz, Pantrop.; 40, (b), JK314.

*Selaginellaceae*

*Selaginella denticulata* (L.) Spring - Chrept, Stenomed.; 197, (b), JK381.

Angiospermae

*Amaryllidaceae*

*Allium flavum* L. subsp. *tauricum* (Besser ex Rchb.) K. Richt. - Gbulb, Eurymed.; JL s.n.; 3, (a), JK25; 20, (a), JK55; 22, (a), JK64.

*Allium vineale* L. - Gbulb, Eurymed.; 26, (a), JK82; 75, (d), JK511.

*Anacardiaceae*

*Cotinus coggygria* Scop. - NP, Med.-Turan.; 96, (a), JK188.

*Pistacia lentiscus* L. - Pcaesp, Stenomed.; 97, (b), JK399.

*Apiaceae*

*Carum graecum* Boiss. & Heldr. subsp. *graecum* - Hscap, Balkan; JL s.n.; 18, (a), JK41; 22, (a), JK69; 23, (a), JK72; 26, (a), JK95; 70, (b), JK305.

*Carum heldreichii* Boiss. – Tscap, Endemic; 110, (b), JK321.

*Conium maculatum* L. - Hscap, Subcosmop.; 101, (b), JK301.

*Daucus carota* L. - Hscap, Subcosmop.; 53, (a), JK155.

*Hellenocarum multiflorum* (Sm.) H. Wolff - Hscap, E-Med.; 25, (a), JK84; 70, (b), Obs.

*Malabaila aurea* (Sm.) Boiss. - Hscap, Balkan; 5, (a), JK31.

*Peucedanum longifolium* Waldst. & Kit. - Hscap, Balkan; 74, (d), JK553.

*Pimpinella tragium* Vill. subsp. *tragium* - Chsuffr, Med.-Turan.; JL s.n.; 308, (a), JK258.

*Prangos ferulacea* (L.) Lindl. - Hscap, NE-Med.-Turan.; 305, (a), JK221.

*Scandix pecten-veneris* L. - Tscap, Subcosmop.; 106, (b), JK441.

*Selinum silaifolium* (Jacq.) Beck - Hscap, SE-Europ.; 1, (a), JK14.

*Seseli parnassicum* Boiss. & Heldr. - Hscap, Endemic; 16, (a), JK48.

*Tordylium apulum* L. - Tscap, Stenomed.; 128, (b), JK475.

*Tordylium officinale* L. - Tscap, E-Med.; 6, (a), JK40.

*Apocynaceae*

*Nerium oleander* L. - Pcaesp, S-Med.; 108, (a), JK109.

*Araceae*

*Arum italicum* Mill. - Grhiz, Stenomed.; 25, (a), JK74; 27, (a), JK87.

*Asparagaceae*

*Bellevalia dubia* (Guss.) Schult. & Schult. f*.* subsp. *boissieri* (Freyn) Feinbrun - Gbulb, C-E-Med.; 344, (a), JK291.

*Leopoldia comosa* (L.) Parl. - Gbulb, C-Med.-Orient.; 26, (a), JK52.

*Ornithogalum montanum* Cirillo - Gbulb, NE-Med.-Mont.; 61, (b), JK318.

*Ornithogalum oligophyllum* E.D. Clarke - Gbulb, Stenomed.-Nordorient.; JL s.n.; 304, (b), JK234.

*Prospero autumnale* (L.) Speta - Gbulb, Eurymed.; 111, (b), JK487.

*Asteraceae*

*Achillea holosericea* Sm. - Hscap, Balkan; JL s.n.; 308, (a), JK300 .

*Achillea ligustica* All. - Hscap, W-Med.; 20, (a), JK70.

*Anthemis chia* L. - Tscap, NE-Med.; 15, (a), JK45; 48, (a), JK181; 52, (a), JK195; 64, (b), JK320.

*Arctium lappa* L. - Hbienn, Euras.; 114, (b), JK395.

*Calendula arvensis* (Vaill.) L.- Tscap, Eurymed.; 115, (a), JK148

*Carduus pycnocephalus* L. - Hbienn, (Eury)Med.-Turan.; 10, (a), JK44

*Carlina lanata* L. - Tscap, Stenomed.; 24, (a), JK77

*Centaurea calcitrapa* L. - Hscap, Subcosmop.; 118, (b), JK442

*Chondrilla juncea* L. - Hscap, Paleotemp.; 119, (a), JK215

*Cirsium arvense* (L.) Scop. - Hscap, Subcosmop.; 14, (a), JK58

*Cirsium candelabrum* Griseb. - Hscap, Balkan; 28, (a), JK78

*Cirsium creticum* (Lam.) d’Urv. - Hscap, NE-Med.; 308, (a), JK260

*Cirsium heldreichii* Halácsy - Hscap, Endemic; 26, (a), JK51

*Crepis foetida* L. - Tscap, Eurymed.; 53, (a), JK99

*Crepis rubra* L. - Tscap, Stenomed.-Nordorient.; 122, (b), JK395

*Dittrichia viscosa* (L.) Greuter - Hscap, Eurymed.; 40, (a), JK115

*Echinops ritro* L. - Hscap, Paleotemp.; ; JL s.n.; 307, (b), JK424

*Filago arvensis* L. - Tscap, Eurymed.-Subsib.; JL s.n.; 304, (b), JK410

*Filago pyramidata* L. - Tscap, Eurymed.; 24, (a), JK62

*Helichrysum plicatum* DC. - Chsuffr, E-Med.-Caucas.; 29, (a), JK90

*Helminthotheca echioides* (L.) Holub - Tscap, Eurymed.; 47, (a), JK108

*Hypochaeris glabra* L. - Tscap, Eurymed.; 52, (a), JK223

*Inula verbascifolia* (Willd.) Hausskn. ssp*. parnassica* (Boiss. & Heldr.) Tutin - Chsuffr, Endemic; 354, (a), JK296

*Lactuca muralis* (L.) Gaertn. - Hscap, Europ-Caucas.; 126, (b), JK340

*Lapsana communis* L. - Tscap, Paleotemp.; 127, (b), JK352

*Notobasis syriaca* (L.) Cass. - Tscap, Stenomed.; 48, (a), JK112

*Onopordum illyricum* L. - Hscap, Eurymed.; 3, (a), JK28; 49, (a), JK110

*Pallenis spinosa* (L.) Cass. - Tscap, Eurymed.; 129, (b), JK325

*Picris rhagadioloides* (L.) Desf. - Tscap, SE-Europ.; 37, (a), JK54

*Pilosella hoppeana* (Schult.) F. W. Schultz & Sch. Bip. - Hros, NE-Med.-Mont.; 16, (a), JK47; 7, (a), JK32; 92, (e), JK995

*Podospermum laciniatum* (L.) DC. - Hbienn, Paleotemp.; 63, (b), JK302

*Pulicaria dysenterica* (L.) Bernh. - Hscap, Eurymed.; 131, (a), JK198

*Reichardia picroides* (L.) Roth - Hscap, Stenomed.; 44, (a), JK107

*Rhagadiolus stellatus* (L.) Gaertn. - Tscap, Eurymed.; 132, (a), JK231

*Senecio vulgaris* L. - Tscap, Cosmop.; 133, (b), JK245

*Sonchus asper* (L.) Hill - Tscap, Euras.; 1, (a), JK15; 48, (a), JK68; 53, (a), JK120

*Sonchus oleraceus* L. - Tscap, Subcosmop.; 41, (f), JK1082

*Taraxacum aleppicum* Dahlst. - Hros, E-Med.; JL s.n.; 316, (a), JK292

*Taraxacum apollinis* Dahlst. - Hros, S-Europ.; JL s.n.; 309, (a), JK254

*Taraxacum gracilens* Dahlst. - Hros, Balkan; JL s.n.; 311, (b), JK402

*Taraxacum mediterraneum* Soest - Hros, Eurymed.; JL s.n.; 307, (a), JK282

*Taraxacum campylodes* G. E. Haglund - Hros, Circumbor.; 135, (a), JK225

*Tussilago farfara* L. - Grhiz, Paleotemp.; 307, (b), JK1440

*Urospermum picroides* (L.) F. W. Schmidt - Tscap, Eurymed.; 136, (b), JK461

*Betulaceae*

*Carpinus orientalis* Mill. - Pcaesp, Pont.; 2, (a), JK19; 45, (a), Obs.; 85, (e), JK767

*Ostrya carpinifolia* Scop. - Pcaesp, Europ-Caucas.; 138, (a), JK1235

*Brassicaceae*

*Alyssum montanum* L. subsp. *montanum* var. *graecum* Halácsy - Chsuffr, Endemic; 6, (b), JK482

*Boraginaceae*

*Alkanna methanea* Hausskn. - Hscap, Endemic; 41, (a), JK125.

*Anchusella variegata* (L.) Bigazzi, Nardi & Selvi - Tscap, Endemic; 200 (b), JK404.

*Asperugo procumbens* L. - Tscap, Paleotemp.; 139, (a), JK235.

*Myosotis alpestris* F. W. Schmidt - Hscap, E-Europ.; 140, (a), JK242.

*Myosotis ramosissima* Rochel - Tscap, Paleotemp.; 26, (a), JK81.

*Myosotis refracta* Boiss. subsp*. refracta* - Tscap, Stenomed.-Orient.; JL s.n.; 308, (a), JK290.

*Campanulaceae*

*Campanula spatulata* Sm. - Grhiz, Balkan; JL s.n.; 1, (a), JK8; 7, (a), JK29; 15, (b), JK310; 52, (a), JK121; 64, (b), JK220.

*Legousia speculum-veneris* (L.) Chaix - Tscap, Eurymed.; JL s.n.; 307, (b), Obs.

*Caprifoliaceae*

*Lonicera implexa* Aiton - Plian, Stenomed.; 45, (b), JK411.

*Caryophyllaceae*

*Herniaria hirsuta* L. - Tscap, Paleotemp.; 35, (a), JK76.

*Herniaria parnassica* Boiss. subsp. *parnassica* - Hcaesp, Balkan; JL s.n.; 28, (a), JK34.

*Minuartia eurytanica* (Boiss. & Heldr.) Hand.-Mazz. - Chsuffr, Endemic; 7, (a), JK42; 75, (d), JK426.

*Petrorhagia glumacea* (Chaub. & Bory) P. W. Ball & Heywood - Tscap, Balkan; 44, (a), JK116.

*Silene radicosa* Boiss. & Heldr. - Hros, Balkan; JL s.n.; 23, (a), JK73.

*Silene roemeri* Friv. - Hros, NE-Med.-Mont.; 75, (d), JK509.

*Celastraceae*

*Euonymus europaeus* L. - NP, Euras.; 155, (a), JK241.

*Chenopodiaceae*

*Chenopodium album* L. - Tscap, Subcosmop.; 62, (a), JK255.

*Clusiaceae*

*Hypericum olympicum* L. - Hscap, Balkan-An.; 75, (d), JK554.

*Hypericum perforatum* L. - Hscap, Subcosmop.; 53, (a), JK119.

*Convolvulaceae*

*Cuscuta epithymum* (L.) L. subsp. *kotschyi* (Desmoulins) Arcangeli - Tpar, Stenomed.; 4, (a), JK20; 5, (a), JK105.

*Cornaceae*

*Cornus mas* L. - Pcaesp, Eurosib.; 158, (b), JK575.

*Crassulaceae*

*Sedum ochroleucum* Chaix - Chsucc, NE-Med.-Mont.; 68, (a), JK161; 90, (f), JK1205.

*Cyperaceae*

*Carex distans* L. - Hcaesp, Eurymed.; 16, (a), JK118.

*Carex macrolepis* DC. - Hcaesp, E-Med.; 8, (a), JK22.

*Carex spicata* Huds. - Hcaesp, Euras.; 8, (a), JK43; 14, (a), JK56.

*Carex vulpina* L. - Hcaesp, Eurosib.; 21, (a), JK157.

*Cyperus longus* L. - Grhiz, Paleotemp.; 49, (a), JK114; 53, (a), JK149

*Eleocharis palustris* (L.) R. Br. - Grhiz, Subcosmop.; 32, (a), JK128; 71, (c), JK504

*Scirpoides holoschoenus* (L.) Soják - Grhiz, Eurymed.; 43, (b), JK327

*Dipsacaceae*

*Dipsacus fullonum* L. - Hbienn, Eurymed.; 160, (d), JK514.

*Knautia integrifolia* (L.) Bertol. - Tscap, Eurymed.; 161, (d), JK535.

*Ericaceae*

*Arbutus andrachne* L. - Pcaesp, E-Stenomed.; 164, (b), JK322.

*Euphorbiaceae*

*Euphorbia herniariifolia* Willd. - Chrept, Balkan-An.; 22, (a), JK53

*Euphorbia myrsinites* L. - Chrept, S-Europ.-Sudsib.; 81, (e), JK754

*Mercurialis annua* L. - Tscap, Paleotemp.; 165, (b), JK392

*Fabaceae*

*Astragalus cylleneus* Fisch. - Chfrut, Endemic; 343, (a), JK200

*Astragalus hamosus* L. - Tscap, Med.-Turan.; 19, (a), JK122

*Bituminaria bituminosa* (L.) C. H. Stirt. - Hscap, Eurymed.; 166, (d), JK541

*Colutea arborescens* L. - Pcaesp, Eurymed.; 16, (a), JK972; 66, (b), JK391

*Genista milli* Heldr. Ex Boiss. – Chfrut, Endemic; 95, (b), JK335

*Hippocrepis comosa* L. - Hcaesp, Centroeurop.; JL s.n.; 304, (b), JK488; 309, (b), JK405.

*Hippocrepis unisiliquosa* L. - Tscap, Eurymed.; 73, (c), JK502.

*Hymenocarpos circinnatus* (L.) Savi - Hscap, Stenomed.; 302, (b), JK429.

*Lathyrus saxatilis* (Vent.) Vis. - Tscap, Eurymed.; 137, (a), JK145.

*Medicago polymorpha* L. - Tscap, Subcosmop.; 302, (b), JK481.

*Medicago sativa* L. - Hscap, Euras.; 302, (b), JK489.

*Melilotus albus* Medik. - Tscap, Subcosmop.; 167, (a), JK285.

*Onobrychis caput-galli* Lam. - Tscap, Stenomed.; 81, (e), JK915; 83, (e), JK985

*Ononis spinosa* L. - Chsuffr, Eurymed.; JL s.n.; 301, (b), JK421.

*Trifolium arvense* L. - Tscap, Euras.-Subozean.-Med.; 87, (e), JK848; 89, (e), JK875.

*Trifolium hirtum* All. - Tscap, Eurymed.; 19, (a), JK117; 75, (d), JK600.

*Trifolium leucanthum* M. Bieb. - Tscap, E-Med.; 12, (a), JK132.

*Trifolium ochroleucon* Huds. - Hcaesp, S-Europ.-Sudsib.; 1, (a), JK10.

*Trifolium pratense* L. - Tscap, Eurosib.; 168, (a), JK146.

*Trifolium strictum* L. - Tscap, Eurymed.; 85, (e), JK855.

*Trifolium tomentosum* L. - Trept, Med.; 5, (a), JK57.

*Vicia canescens* Labill. - E-Med; 91, (e), JK775.

*Vicia lathyroides* L. - Tscap, Eurymed.; JL s.n.; 308, (a), JK246.

*Fagaceae*

*Quercus ilex* L. - Pscap, Stenomed.; 169, (b), JK304.

*Gentianaceae*

*Blackstonia perfoliata* (L.) Huds. subsp. *perfoliata* - Tscap, Eurymed.; 170, (b), JK445.

*Centaurium erythraea* Rafn subsp. *erythraea* - Hbienn, Paleotemp.; 171, (a), JK295.

*Geraniaceae*

*Geranium molle L.* subsp. *molle* - Tscap, Subcosmop.; JL s.n.; 311, (b), JK474.

*Geranium purpureum* Vill. - Tscap, Eurymed.; 16, (a), JK18; 67, (b), JK448.

*Iridaceae*

*Iris unguicularis* Poirret subsp. *carica* (W. Schulze) Davis & Jury var. *angustifolia* (Boiss. & Heldr.) Davis & Jury - Grhiz, Endemic; 109, (a), JK228.

*Juncaceae*

*Juncus articulatus* L. - Grhiz, Circumbor.; 3, (a), JK29; 8, (a), JK59; 58, (b), JK408

*Juncus effusus* L. - Hcaesp, Cosmop.; 40, (a), JK111; 49, (a), JK103.

*Juncus hybridus* Brot. - Hcaesp, Eurymed.; 352, (a), JK251.

*Juncus inflexus* L. - Hcaesp, Paleotemp.; JL s.n.; 8, (a), JK92.

*Luzula campestris* (L.) DC. - Hcaesp, Europ-Caucas.; 67, (b), JK464.

*Lamiaceae*

*Lamium amplexicaule* L. - Tscap, Paleotemp.; 309, (a), JK239.

*Marrubium vulgare* L. - Hscap, Subcosmop.; 173, (b), JK477.

*Mentha longifolia* (L.) L. - Hscap, Submed.-Euras.; 14, (a), JK11; 53, (a), JK141; 58, (b), JK406

*Origanum vulgare* L. subsp. *viridulum* (Martrin-Donos) Nyman - Hscap, E-Med.-Asiat.; 174, (b), JK428.

*Scutellaria rupestris* Boiss. & Heldr. subsp. *adenotricha* (Boiss. & Heldr.) Greuter & Burdet - Hscap, Balkan; 6, (a), JK129; 18, (a), JK106.

*Sideritis clandestina* (Bory & Chaub.) Hayek subsp. *peloponnesiaca* (Boiss. & Heldr.) Baden - Chsuffr, Endemic; 93, (b), JK485.

*Linaceae*

*Linum bienne* Miller - Hscap, Med.-Atl.; 53, (a), JK50.

*Linum strictum* L. - Tscap, Stenomed.; 82, (e), JK952.

*Liliaceae*

*Gagea pusilla* (F. W. Schmidt) Sweet - Gbulb, Europ.-S-Sib.; JL s.n.; 305, (a), JK275.

*Loranthaceae*

*Loranthus europaeus* Jacq. - Pep, Europ.-Caucas.; 180, (b), JK462.

*Viscum album* L. – Pep, Euras.; 82, (e), JK1474.

*Malvaceae*

*Malva sylvestris* L. - Hscap, Subcosmop.; 181, (b), JK414.

*Oleaceae*

*Fraxinus ornus* L. - Pscap, Eurymed.; 182, (b), JK425.

*Orchidaceae*

*Anacamptis pyramidalis* (L.) Rich. - Hscap, Eurymed.; 183, (b), JK447.

*Epipactis helleborine* (L.) Crantz - Grhiz, Submed.-Euras.; 1, (a), JK2.

*Plantaginaceae*

*Plantago lagopus* L. - Tscap, Eurymed.; 49, (a), JK269.

*Plantago lanceolata* L. - Hros, Cosmop.; JL s.n.; 301, (b), JK478.

*Plantago major* L. - Hros, Subcosmop.; 21, (a), JK91.

*Plantago weldenii* Rchb. - Tscap, Eurymed.; 14, (a), JK1269.

*Poaceae*

*Aegilops biuncialis* Vis. - Tscap, Stenomed.-Orient.; JL s.n. 307, (b), JK500.

*Aegilops neglecta* Bertol. - Tscap, Med.-Turan.; 17, (a), JK88; 43, (b), JK401; 51, (a), JK135; 55, (b), JK415.

*Anisantha madritensis* (L.) Nevski - Tscap, Eurymed.; 128, (b), JK348.

*Anisantha sterilis* (L.) Nevski - Tscap, Eurymed.-Turan.; JL s.n.; 6, (a), JK182; 19, (a), JK60.

*Anisantha tectorum* (L.) Nevski - Tscap, Paleotemp.; JL s.n.; 20, (a), JK46; 90, (f), JK1125.

*Avena sterilis* L. - Tscap, Med.-Turan.; 41, (b), JK480; 76, (e), JK882; 81, (e), JK890; 84, (e), JK905.

*Briza media* L. - Hcaesp, Eurosib.; 46, (b), JK443.

*Brizochloa humilis* (M. Bieb.) Chrtek & Hadac - Tcaesp, E-Med.; 74, (d), JK598.

*Bromopsis riparia* (Rehmann) Holub - Hcaesp, Eurymed.-Orient.; 4, (a), JK144.

*Bromus hordeaceus* subsp. *molliformis* (Billot) Maire & Weiller - Tscap, S-Europ.; JL s.n.; 301, (b), JK457; 308, (b), JK595.

*Bromus intermedius* Guss. - Tscap, Eurymed.; JL s.n.; 315, (b), JK546.

*Bromus japonicus* Thunb. - Tscap, Paleotemp.; 19, (a), JK61.

*Bromus scoparius* L. - Tscap, Stenomed.; 20, (a), JK134.

*Dasypyrum villosum* (L.) P. Candargy - Tscap, Eurymed.-Turan.; 48, (a), JK98.

*Festuca jeanpertii* (St.-Yves) Markgr. subsp. *jeanpertii* - Hcaesp, Eurymed.; 1, (a), JK6; 3, (a), JK85; 74, (d), JK582; 83, (e), JK5825.

*Helictotrichon agropyroides* (Boiss.) Henrand - Hcaesp, Endemic; 312, (b), JK455.

*Holcus lanatus* L. - Hcaesp, Circumbor.; 188, (b), JK308.

*Koeleria splendens* C. Presl - Hcaesp, Balkan-It.; 92, (b), JK382.

*Lagurus ovatus* L. - Tscap, Eurymed.; 189, (a), JK174.

*Piptatherum miliaceum* (L.) Coss. - Hcaesp, Eurymed.-Turan.; 49, (a), JK126.

*Ochlopoa annua* (L.) H. Scholz - Tcaesp, Cosmop.; 190, (b), JK384.

*Poa timoleontis* Boiss. - Hcaesp, Stenomed.-Orient.; JL s.n.; 304, (b), JK412.

*Schedonorus arundinaceus* (Schreb.) Dumort. - Hcaesp, Paleotemp.; 307, (b), JK420.

*Sesleria vaginalis* Boiss. & Orph - Hcaesp, Endemic; 305, (a), JK281.

*Stipa pulcherrima* K. Koch subsp. *epilosa* (Martinovský) Tzvelev - Hcaesp, Balkan; 303, (a), JK252; 308, (a), JK299.

*Trachynia distachya* (L.) Link - Tscap, Med.-Turan.; 41, (a), JK101.

*Trisetum flavescens* (L.) P.Beauv. subsp. *tenue* (Formánek) Strid - Hcaesp, Stenomed.; 7, (a), JK49.

*Polygonaceae*

*Rumex crispus* L. - Hscap, Subcosmop.; 8, (a), JK159.

*Primulaceae*

*Anagallis arvensis* L. - Trept, Subcosmop.; 192, (b), JK450.

*Cyclamen hederifolium* Aiton - Gbulb, Eurymed.; 67, (b), JK341.

*Primula acaulis* (L.) L. - Hros, Europ-Caucas.; 201, (b), JK479.

*Primula veris* L. subsp. *columnae* (Ten.) Lüdi - Hros, NE-Med.-Mont.; JL s.n.; 308, (a), JK284.

*Samolus valerandi* L. - Hscap, Subcosmop.; 201, (b), JK431.

*Ranunculaceae*

*Anemone coronaria* L. - Gbulb, Eurymed.-Orient.; 135, (a), JK210.

*Rosaceae*

*Fragaria vesca* L. - Hrept, Eurosib.; 85, (e), JK1102.

*Potentilla reptans* L. - Hros, Paleotemp.; 14, (a), JK458; 21, (a), JK71.

*Potentilla speciosa* Willd. - Chpulv, Balkan-An.; 69, (b), JK460.

*Prunus mahaleb* L. - Pcaesp, S-Europ.-Pont.; 55, (b), JK468.

*Prunus spinosa* L. - Pcaesp, Europ-Caucas.; 48, (a), JK102.

*Rubus sanctus* Schreb. - NP, Eurymed.; 38, (b), JK355; 45, (a), JK38; 47, (a), JK35.

*Sorbus umbellata* (Desf.) Fritsch - MP, E-Med.; 23, (a), JK79.

*Rubiaceae*

*Asperula arvensis* L. - Tscap, Med.-Submed.; 11, (a), JK11.

*Asperula chlorantha* (Boiss. & Heldr.) Hayek - Hscap, Balkan; 25, (a), JK150; 69, (b), JK418.

*Cruciata laevipes* Opiz - Hscap, Euras.; 5, (a), JK7; 11, (a), JK192.

*Cruciata pedemontana* (Bellardi) Ehrend. - Tscap, Eurymed.; JL s.n.; 300, (b), JK343.

*Galium rotundifolium* L. - Hscap, Orof.-W-Eurasiat.; 85, (e), JK782.

*Salicaceae*

*Salix alba* L. - Pscap, E-Med.-Mont.; 49, (a), JK143; 53, (a), JK175.

*Salix elaeagnos* Scop. - Pscap, Orof.-S-Europ.; 202, (b), JK375.

*Scrophulariaceae*

*Digitalis laevigata* Waldst. & Kit. subsp. *graeca* (Ivanina) H. Werner - Hscap, Balkan; 302, (b), JK525.

*Parentucellia latifolia* (L.) Caruel - Tscap, Med.-Atl.; 196, (a), JK287.

*Verbascum sinuatum* L. - Hbienn, Eurymed.; 135, (a), JK240.

*Verbascum undulatum* Lam. - Hscap, Balkan; 135, (a), JK214.

*Veronica anagallis-aquatica* L. - Hscap, Cosmop.; 71, (c), JK545.

*Veronica hederifolia* L. - Tscap, Euras.; JL s.n.; 300, (b), JK440.

*Veronica praecox* All. - Tscap, C-Europ.-Submed.; JL s.n.; 309, (a), JK202.

*Solanaceae*

*Solanum nigrum* L. - Tscap, Cosmop.; 198, (b), JK388.

*Valerianaceae*

*Centranthus ruber* (L.) DC. - Chsuffr, Stenomed.; 74, (d), JK505.

*Valeriana tuberosa* L. - Hscap, Eurymed.; 4, (a), JK178.

*Verbenaceae*

*Verbena officinalis* L. - Hscap, Cosmop.; 200, (b), JK422.

*Vincaceae*

*Vinca major* L. - Chrept, Eurymed.; 1, (a), JK4.

*Violaceae*

*Viola kitaibeliana* Schultes - Tscap, Europ-Caucas.; 318, (a), JK274.

*Viola reichenbachiana* Jordan ex Boreau – Hscap, Eurosib.; 316, (b), JK257.
