# Supplementary material for: The native flora of Mountain Panachaikon (Peloponnese, Greece): new records and diversity
Source: J Biol Res (Thessalon). 2014 Jun 3;21(1):9. doi: 10.1186/2241-5793-21-9 (PMC4389996; doi:10.1186/2241-5793-21-9)
Supplement: Supplementary file 2 — Additional file 2: Protection status and extinction risk designation of the Greek endemic taxa of Mt. Panachaikon and their geographical distribution in the floristic regions of Greece. (DOC 232 KB) [file 40709_2013_7_MOESM2_ESM.doc]

**Additional file 2. Protection status and extinction risk designation of the Greek endemic taxa of Mt. Panachaikon and their geographical distribution in the floristic regions of Greece. (IoI: Ionian islands, StE: Sterea Ellada, Pe: Peloponnese, Kik: Cyclades, KK: Crete, Karpathos, EAe: East Aegean islands, NPi: North Pindos, SPi: South Pindos, EC: East Central Greece, NC: North Central Greece, NE: North-East Greece, NAe: North Aegean islands, WAe: West Aegean islands).**

| **Family** | **Greek endemic taxa** | **Protection /**  **Extinction risk status** | **Pe** | **StE** | **WAe** | **IoI** | **SPi** | **NPi** | **EC** | **NC** | **NE** | **NAe** | **Kik** | **KK** | **EAe** |
| --- | --- | --- | --- | --- | --- | --- | --- | --- | --- | --- | --- | --- | --- | --- | --- |
| **Pinaceae** | *Abies cephalonica* Loudon | - / IUCN_c_ (LC) | **1** | **1** | **1** | **1** | **1** |  |  | **1** |  |  |  |  |  |
| **Amarylidaceae** | *Allium achaium* Boiss. & Orph. | -/- | **1** | **1** |  |  | **1** | **1** |  |  |  |  |  |  |  |
| **Amarylidaceae** | *Allium frigidum* Boiss. & Heldr. | PD 67/81 / IUCN_a_ (R), ERL (DD) | **1** |  |  |  |  |  |  |  |  |  |  |  |  |
| **Apiaceae** | *Carum heldreichii* Boiss. | -/- | **1*** | **1** |  |  |  |  |  |  |  |  |  |  |  |
| **Apiaceae** | *Geocaryum parnassicum* (Boiss. & Heldr.) Engstr. | -/- | **1** | **1** | **1** |  |  |  |  |  |  |  |  |  |  |
| **Apiaceae** | *Geocaryum peloponnesiacum* Engstrand | -/- | **1** |  |  | **1** |  |  |  |  |  |  |  |  |  |
| **Apiaceae** | *Peucedanum achaicum* Halacsy | PD 67/81 / IUCN_b_ (R), RDB_i_ (VU) | **1** |  |  |  |  |  |  |  |  |  |  |  |  |
| **Apiaceae** | *Seseli parnassicum* Boiss. & Heldr. | PD 67/81 / IUCN_b_ (R) | **1*** | **1** |  |  | **1** |  |  |  |  |  |  |  |  |
| **Apiaceae** | *Trinia frigida* (Boiss. & Heldr.) Drude | -/- | **1** | **1** |  | **?** |  |  |  |  |  |  |  |  |  |
| **Araceae** | *Biarum tenuifolium* (L.) Schott subsp. *abbreviatum* (Schott) Richter | -/- | **1** | **1** |  | **1** | **1** |  |  |  |  |  |  |  |  |
| **Asparagaceae** | *Muscari pulchellum* Heldr. & Sart. | -/- | **1** | **1** | **1** |  |  |  |  |  |  |  | **1** |  |  |
| **Asparagaceae** | *Ornithogalum fimbriatum* Willd. subsp. *gracilipes* (Zahar.) Landström | -/- | **1** | **1** |  |  |  |  |  |  |  |  |  |  |  |
| **Asteraceae** | *Achillea umbellata* Sm. | -/- | **1** | **1** | **1** |  |  |  |  |  |  |  |  |  |  |
| **Asteraceae** | *Anthemis brachmannii* Boiss. & Heldr. | PD 67/81 / IUCN_a_ (K) | **1** |  |  |  |  |  |  |  |  |  |  |  |  |
| **Asteraceae** | *Centaurea affinis* Friv. subsp. *laconiae* Prodan | -/- | **1** | **1** |  |  |  |  |  |  |  |  |  |  |  |
| **Asteraceae** | *Centaurea raphanina* Sm. subsp. *mixta* (DC.) Runemark | -/ IUCN_a_ (*nt*) | **1** | **1** | **1** |  |  |  |  |  |  |  | **1** |  | **1** |
| **Asteraceae** | *Cirsium heldreichii* Halácsy | PD 67/81 / IUCN_a_ (K) | **1*** | **1** | **1** |  | **1** | **1** |  |  |  |  |  |  |  |
| **Asteraceae** | *Cirsium hypopsilum* Boiss. & Heldr. | -/ IUCN_a_ (nt) | **1** |  |  |  |  |  |  |  |  |  |  |  |  |
| **Asteraceae** | *Crepis hellenica* Kamari subsp. *hellenica* | -/ IUCN_a_ (*nt*) | **1** | **1** | **1** |  | **1** |  | **1** |  | **?** |  | **1** |  | **1** |
| **Asteraceae** | *Crepis incana* Sm. | -/ IUCN_a_ (nt) | **1** | **1** | **1** |  |  |  |  |  |  |  |  |  |  |
| **Asteraceae** | *Inula verbascifolia* (Willd.) Hausskn. subsp. *parnassica* (Boiss. & Heldr.) Tutin | -/- | **1** | **1** | **1** |  |  |  |  |  |  |  |  |  |  |
| **Asteraceae** | *Leontodon graecus* Boiss. & Heldr. | PD 67/81 /- | **1** | **1** | **1** | **1** |  |  |  | **?** |  | **1** | **1** |  | **1** |
| **Asteraceae** | *Scorzonera crocifolia* Sm. | PD 67/81 / IUCN_a_ (nt) | **1** | **1** | **1** | **1** | **1** |  | **1** | **?** |  | **1** | **1** |  |  |
| **Asteraceae** | *Tragopogon brevirostris* DC. subsp. *longifolius* (Heldr. & Sart. ex Boiss.) I. B. K. Richardson | -/- | **1** | **1** | **1** |  |  |  |  |  |  |  |  |  |  |
| **Berberidaceae** | *Gymnospermium altaicum* (Pall.) Spach subsp. *peloponnesiacum* Phitos | -/ IUCN_b_ (E), RDB_ii_ (NT) | **1** |  |  |  |  |  |  |  |  |  |  |  |  |
| **Boraginaceae** | *Alkanna methanea* Hausskn. | PD 67/81 / IUCN_a_ (I) | **1** | **1** |  |  |  |  |  |  |  |  |  |  |  |
| **Boraginaceae** | *Anchusella variegata* (L.) Bigazzi, Nardi & Selvi | -/- | **1** | **1** | **1** | **1** |  |  | **1** | **?** |  | **1** | **1** | **1** | **1** |
| **Boraginaceae** | *Onosma erecta* Sm. subsp. *malickyi* Teppner | -/ IUCN_a_ (*nt*) | **1** |  |  |  |  |  |  |  |  |  |  |  |  |
| **Boraginaceae** | *Onosma kaheirei* Teppner | -/- | **1** | **1** | **1** |  |  |  |  | **1** |  |  |  |  | **1** |
| **Brassicaceae** | *Alyssum montanum* L. subsp*. montanum* var*. graecum* Halacsy | -/- | **1*** | **1** | **1** |  |  | **1** | **1** | **1** |  | **1** |  |  |  |
| **Brassicaceae** | *Arabis subflava* B. M.G. Jones | PD 67/81 / IUCN_b_ (R) | **1** | **1** |  |  |  |  |  |  |  |  |  |  |  |
| **Brassicaceae** | *Aurinia moreana* Tzanoud. & Iatrou | -/- | **1** |  |  |  |  |  |  |  |  |  |  |  |  |
| **Brassicaceae** | *Draba lacaitae* Boiss. | -/ IUCN_a_ (nt) | **1** | **1** | **1** |  |  |  |  |  |  |  |  |  |  |
| **Brassicaceae** | *Erysimum asperulum* Boiss. & Heldr. | -/- | **1** | **1** |  |  |  |  | **1** |  |  |  |  |  |  |
| **Brassicaceae** | *Erysimum cephalonicum* Polatschek | -/- | **1** | **1** |  | **1** | **1** | **1** |  | **1** |  |  |  |  |  |
| **Brassicaceae** | *Erysimum graecum* Boiss & Heldr | -/- | **1** | **1** | **1** |  |  |  | **1** |  |  |  |  |  |  |
| **Brassicaceae** | *Erysimum pectinatum* Bory & Chaub. | PD 67/81 / IUCN_a_ (nt) | **1** |  |  |  |  |  |  |  |  |  |  |  |  |
| **Brassicaceae** | *Erysimum pusillum* Bory & Chaub. | -/- | **1** | **1** |  |  |  |  |  |  |  |  |  |  |  |
| **Brassicaceae** | *Malcolmia graeca* Boiss. & Spruner subsp. *bicolor* (Boiss. & Heldr.) Stork | -/- | **1** | **1** |  | **1** |  |  |  |  |  |  |  |  |  |
| **Brassicaceae** | *Thlaspi graecum* Jordan | -/ IUCN_a_ (nt) | **1** |  |  |  |  |  |  |  |  |  |  |  |  |
| **Campanulaceae** | *Campanula radicosa* Bory & Chaub. | -/ IUCN_a_ (nt) | **1** | **1** |  |  |  |  |  |  |  |  |  |  |  |
| **Campanulaceae** | *Edraianthus parnassicus* (Boiss. & Spruner) Halacsy | -/- | **1** | **1** |  |  |  |  |  |  |  |  |  |  |  |
| **Caryophyllaceae** | *Arenaria guicciardii* Heldr. ex Boiss. | PD 67/81 / IUCN_b_ (R) | **1** | **1** |  | **1** |  |  |  |  |  |  |  | **1** | **1** |
| **Caryophyllaceae** | *Cerastium candidissimum* Correns | -/ IUCN_a_ (nt) | **1** | **1** | **1** | **1** | **1** | **1** | **1** |  |  |  |  |  |  |
| **Caryophyllaceae** | *Cerastium illyricum* Ard. subsp. *brachiatum* (Lonsing) Jalas | -/- | **1** |  |  | **1** |  |  |  |  |  |  |  |  |  |
| **Caryophyllaceae** | *Dianthus androsaceus* (Boiss. & Heldr.) Hayek | -/ RDB_i_ (R) | **1** |  |  |  |  |  |  |  |  |  |  |  |  |
| **Caryophyllaceae** | *Dianthus biflorus* Sm. | -/ IUCN_a_ (nt) | **1** | **1** | **1** |  | **1** | **1** | **1** |  |  |  |  |  |  |
| **Caryophyllaceae** | *Dianthus serratifolius* Sm. subsp. *serratifolius* | -/ IUCN_a_ (nt) | **1** | **1** |  |  |  |  |  |  |  |  |  |  |  |
| **Caryophyllaceae** | *Dianthus tymphresteus* (Boiss. & Spruner) Heldr. & Sart. | -/- | **1** | **1** |  |  |  |  |  |  |  |  |  |  |  |
| **Caryophyllaceae** | *Minuartia confusa* (Boiss.) Maire & Petitm. | -/- | **1** | **1** |  |  |  |  |  |  |  |  |  |  |  |
| **Caryophyllaceae** | *Minuartia eurytanica* (Boiss. & Heldr.) Hand.-Mazz. | -/ IUCN_a_ (nt) | **1*** | **1** |  |  | **1** | **1** |  | **1** |  |  |  |  | **1** |
| **Caryophyllaceae** | *Petrorhagia graminea* (Sm.) P.W.Ball & Heywood | -/ IUCN_a_ (nt) | **1** |  |  | **1** | **1** |  |  |  |  |  |  |  |  |
| **Caryophyllaceae** | *Silene auriculata* Sm. | -/- | **1** | **1** |  |  |  |  |  |  |  |  |  |  |  |
| **Caryophyllaceae** | *Silene congesta* Sm. | -/ IUCN_a_ (nt) | **1** | **1** |  |  | **1** |  |  |  |  |  |  |  |  |
| **Caryophyllaceae** | *Silene gigantea* (L.) L. subsp. *hellenica* Greuter | -/- | **1** | **1** | **1** |  |  |  |  |  |  |  |  |  |  |
| **Caryophyllaceae** | *Silene italica* (L.) Pers. subsp. *peloponnesiaca* Greuter | -/- | **1** | **1** |  |  |  |  |  |  |  |  |  |  |  |
| **Colchicaceae** | *Colchicum graecum* K. Perss. | -/- | **1** | **1** |  |  | **1** | **1** |  |  |  |  |  |  |  |
| **Crassulaceae** | *Sedum laconicum* Boiss. & Heldr. subsp. *laconicum* | -/- | **1** | **1** | **1** | **1** | **1** | **1** | **1** |  | **1** |  |  | **1** |  |
| **Dipsacaceae** | *Pterocephalus perennis* Coulter subsp. *perennis* | -/- | **1** | **1** | **1** |  |  |  | **1** | **1** | **1** |  |  |  |  |
| **Fabaceae** | *Astragalus cylleneus* Fisch. | -/- | **1** |  |  |  |  |  |  |  |  |  |  |  |  |
| **Fabaceae** | *Genista milii* Heldr. ex Boiss. | PD 67/81 / IUCN_b_ (R) | **1*** | **1** | **1** |  |  |  |  |  |  |  |  |  |  |
| **Fabaceae** | *Melilotus graecus* (Boiss. & Spruner) Lassen | -/ IUCN_a_ (nt) | **1** | **1** |  | **1** | **1** | **1** | **1** |  |  |  |  | **1** |  |
| **Fabaceae** | *Onobrychis ebenoides* Boiss. & Spruner | PD 67/81 / IUCN_a_ (nt) | **1** | **1** | **1** |  |  |  |  |  |  |  |  |  | **1** |
| **Fabaceae** | *Trifolium aurantiacum* Boiss. & Spruner | -/ IUCN_a_ (nt) | **1** | **1** |  |  |  | **1** |  |  |  |  |  |  |  |
| **Fabaceae** | *Trifolium parnassi* Βοiss. & Spruner | -/ IUCN_a_ (nt) | **1** | **1** |  |  | **1** | **1** |  | **1** |  |  |  |  |  |
| **Geraniaceae** | *Erodium chrysanthum* L'Her. ex DC. | Bern, PD 67/81 / IUCN_b_ (VU) | **1** | **1** |  |  |  |  |  |  |  |  |  |  |  |
| **Iridaceae** | *Crocus boryi* Gay | -/ IUCN_a_ (nt) | **1** | **1** |  | **1** |  |  |  |  |  |  |  | **1** | **1** |
| **Iridaceae** | *Crocus hadriaticus* Herb. | -/ IUCN_a_ (nt) | **1** | **1** |  | **1** | **1** | **1** |  |  |  |  |  |  |  |
| **Iridaceae** | *Iris unguicularis* Poiret subsp. *carica* (W. Schulze) Davis & Jury var. *angustifolia* (Boiss. & Heldr.) Davis & Jury | -/- | **1** | **1** | **1** | **1** |  |  |  |  |  |  | **1** |  |  |
| **Lamiaceae** | *Marrubium cylleneum* Boiss. & Heldr. | -/- | **1** |  |  |  |  |  |  |  |  |  |  |  |  |
| **Lamiaceae** | *Marrubium velutinum* Sm. | -/ IUCN_a_ (nt) | **1** | **1** |  |  | **1** | **1** |  | **1** |  |  |  |  |  |
| **Lamiaceae** | *Scutellaria rupestris* Boiss. & Heldr. subsp. *parnassica* (Boiss. & Heldr.) Greuter & Burdet | PD 67/81 / IUCN_b_ (*R*) | **1** | **1** | **1** |  |  |  | **1** |  |  |  |  |  |  |
| **Lamiaceae** | *Sideritis clandestina* (Bory & Chaub.) Hayek subsp. *peloponnesiaca* (Boiss. & Heldr.) Baden | PD 67/81 / IUCN_a_ (*nt*) | **1** |  |  |  |  |  |  |  |  |  |  |  |  |
| **Lamiaceae** | *Stachys graeca* Boiss. & Heldr. | -/- | **1** | **1** | **1** |  | **1** |  |  | **1** |  |  |  |  |  |
| **Liliaceae** | *Fritillaria graeca* Boiss. & Spruner var. *guicciardii* (Heldr. & Sart.) Boiss. | Bern / IUCN_c_ (DD), ERL (DD) | **1** | **1** | **1** |  |  |  |  |  |  |  |  |  |  |
| **Liliaceae** | *Fritillaria mutabilis* Kamari | -/- | **1** | **1** |  | **1** | **1** |  |  |  |  |  |  |  |  |
| **Orchidaceae** | *Ophrys argolica* H. Fleischm. | CITES, Bern, PD 67/81, 92/43/EEC / IUCN_c_ (VU) , ERL (VU), RDB_ii_ (VU), | **1** | **1** |  |  |  |  |  |  |  |  |  |  |  |
| **Plantaginaceae** | *Plantago atrata* Hoppe subsp. *graeca* (Halacsy) Holub | -/- | **1** | **1** |  |  | **1** | **1** |  |  |  |  |  |  |  |
| **Ranunculaceae** | *Nigella arvensis* L. subsp. *aristata* (Sm.) Nyman | -/- | **1** | **1** | **1** | **1** | **1** | **1** | **1** | **1** | **?** |  |  |  |  |
| **Poaceae** | *Festuca jeanpertii* (St-Yves) Markgr. subsp. *achaica* (Markgr.-Dann.) Markgr.-Dann. | -/- | **1** |  |  |  |  |  |  |  |  |  |  |  |  |
| **Poaceae** | *Helictotrichon agropyroides* (Boiss.) Henrand | -/- | **1** | **1** | **1** |  |  |  |  |  |  |  | **1** | **1** |  |
| **Poaceae** | *Helictotrichon convolutum* (C. Presl) Henrard subsp. *heldreichii* (Parl.) Gervais | -/- | **1** | **1** | **1** |  |  |  |  |  |  |  |  |  |  |
| **Poaceae** | *Sesleria vaginalis* Boiss. & Orph. | PD 67/81 / IUCN_a_ (K) | **1** | **1** | **1** |  | **1** | **1** |  | **1** |  |  |  |  |  |
| **Rubiaceae** | *Asperula aristata* L. subsp. *thessala* (Boiss. & Heldr.) Hayek | -/- | **1** | **1** | **1** |  | **1** | **1** | **1** | **1** | **1** | **1** |  |  |  |
| **Rubiaceae** | *Asperula lutea* Sm. | PD 67/81 / IUCN_a_ (K) | **1** | **1** |  |  |  |  |  |  |  |  |  | **?** |  |
| **Rubiaceae** | *Galium melanantherum* Boiss. | -/ IUCN_a_ (nt) | **1** | **1** | **1** |  |  |  |  |  |  |  | **1** |  |  |
| **Rubiaceae** | *Galium peloponnesiacum* Erhend. & Krendl | -/ IUCN_a_ (nt) | **1** |  |  | **1** |  |  |  |  |  |  |  | **1** |  |
| **Rubiaceae** | *Galium taygeteum* Κrendl | -/- | **1** |  |  |  |  |  |  |  |  |  |  |  |  |
| **Rubiaceae** | *Galium violaceum* Krendl | -/- | **1** |  |  |  |  |  |  |  |  |  |  |  |  |
| **Scrophulariaceae** | *Scrophularia heterophylla* Willd. var. *heterophylla* | -/- | **1** | **1** | **1** | **1** |  |  | **1** |  | **1** | **1** | **1** | **1** | **1** |
| **Scrophulariaceae** | *Verbascum epixanthinum* Boiss. & Heldr. | -/- | **1** | **1** | **1** |  | **1** | **1** |  | **1** |  |  |  |  |  |
| **Scrophulariaceae** | *Veronica chamaedrys* L. subsp. *chamaedryoides* (Bory & Chaub.) M. A. Fisch. | -/- | **1** | **1** | **1** | **1** | **1** | **1** | **1** | **1** |  | **1** |  |  |  |
| **Scrophulariaceae** | *Veronica glauca* Sm. subsp. *chaubardii* (Boiss. & Reuter) Maire & Petitm. | -/- | **1** |  |  | **1** |  |  |  |  |  |  |  |  |  |
| **Scrophulariaceae** | *Veronica glauca* Sm. subsp. *peloponnesiaca* (Boiss. & Orph.) Maire & Petitm. | -/- | **1** | **1** | **1** | **1** | **1** | **1** |  | **1** | **1** |  | **1** |  |  |
| **Violaceae** | *Viola phitosiana* Erben | -/- | **1** | **1** | **1** |  | **1** | **1** | **1** | **1** | **1** |  |  |  |  |

Symbols and Abbreviations:

1 : the taxon is present.

* : new record for Pe.

? : the taxon is doubtfully present.

CITES: Convention on International Trade in Endangered Species of Wild Fauna and Flora [33].

Bern: Convention on the conservation of European Wildlife and Natural Habitats [35].

PD 67/81: Greek Presidential Decree 67/1981 on the protection of the native flora and wild fauna of Greece [36].

92/43/EEC: European Council Directive 92/43/EEC [34].

IUCN_X_: Red Lists of Threatened Plants (a: [37], b: [39], c: [38]).

ERL: European Red List of Vascular Plants [40].

RDB_y_: Red Data Book of Rare and Threatened Plants of Greece (i: [27], ii: [28]).

IUCN extinction risk categories: Neihter rare nor threatened (nt), Least Concern (LC), Data Deficient (DD), Insufficiently Known (K), Indeterminate (I), Rare (R), Near Threatened (NT), Vulnerable (VU), Endangered (E); italics indicate evaluation at species level.
